# Development of Lewis-Sumner Syndrome or Multifocal Acquired Demyelinating Sensory and Motor Neuropathy (MADSAM) Following COVID-19 Infection

**DOI:** 10.7759/cureus.46643

**Published:** 2023-10-07

**Authors:** Brandon L Welborn, Jeff Benjamin

**Affiliations:** 1 Neurology, Edward Via College of Osteopathic Medicine, Spartanburg, USA; 2 Neurology, Bon Secours Diane Collins Neuroscience Institute, Greenville, USA

**Keywords:** lewis-sumner syndrome, neuropathy, atypical cidp, madsam, covid-19

## Abstract

Multifocal acquired demyelinating sensory and motor neuropathy (MADSAM), also known as asymmetrical or multifocal chronic inflammatory demyelinating polyneuropathy (CIDP) or Lewis-Sumner syndrome, is a painless asymmetric demyelinating sensorimotor mononeuropathy multiplex and is an atypical and rare variant of CIDP. We report a case of a 48-year-old female who presented with complaints of shortness of breath, fatigue, difficulty walking and speaking, and primarily right unilateral symptoms involving multiple peripheral nerves, the right phrenic nerve, and the facial nerve following a coronavirus disease 2019 (COVID-19) infection. She was diagnosed with MADSAM after an extensive physical examination, electromyography (EMG), nerve conduction tests, and laboratory testing. She improved following the initiation of long-term treatment with intravenous immunoglobulin (IVIG). To the best of our knowledge, this is the second reported case of MADSAM following a COVID-19 infection. There have been reports of demyelinating diseases of the central and peripheral nervous systems related to COVID-19; however, it remains unclear whether COVID-19 is the causative agent or only a correlative.

## Introduction

Since the onset of the coronavirus disease 2019 (COVID-19) pandemic, numerous case reports have noted links between the virus and the development of autoimmune diseases [1]. Since 2020, all reported cases of COVID-19 neuropathy, whether virus- or vaccine-related, have been Guillain-Barré syndrome, typical chronic inflammatory demyelinating polyneuropathy (CIDP), or Miller-Fisher syndrome-like, with one case describing the virus's association with multifocal acquired demyelinating sensory and motor neuropathy (MADSAM) [2]. MADSAM is relatively rare, accounting for 5-10% of CIDP cases with a prevalence of one to nine cases per 1,000,000 people, while typical CIDP has a prevalence of 4.8-8.9 cases per 100,000 people [3,4]. No antecedent infections or vaccinations have been consistently linked to the development of CIDP, but they have preceded approximately 10% of cases, suggesting a pathophysiological link; however, this link remains unproven [5]. MADSAM is a dysimmune motor and sensory peripheral neuropathy that typically manifests as a chronic sensorimotor mononeuropathy multiplex with an asymmetrical, insidious onset, and slow progression, affecting primarily the distal and intermediate nerve segments [6]. This disease initially involves single nerves in the upper extremities, spreads to the lower extremities, and can eventually progress to symmetrical involvement, mimicking typical CIDP [7]. Multifocal, persistent conduction blocks are also a common finding in this disease [7]. This case is a unique presentation of MADSAM and strengthens the case for the emergence of COVID-19-associated CIDP-like neuropathies.

## Case presentation

**Figure 1:**
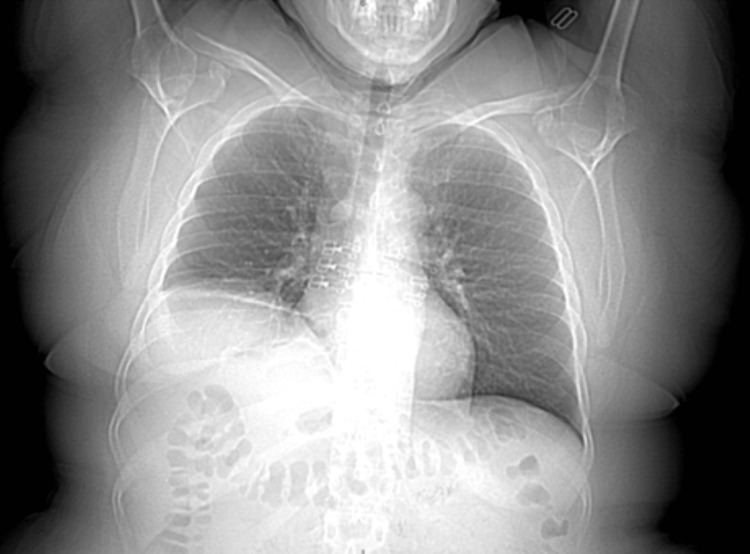
Coronal chest x-ray performed upon initial presentation to the ED showing right diaphragm paralysis.

A 48-year-old female was consulted by outpatient neurology following a visit to the emergency department (ED) one day prior with progressively deteriorating shortness of breath (SOB), muscle fatigue and weakness, and slurred speech. She was diagnosed with COVID-19 eight months prior to presenting to the ED. Her current symptoms started eight weeks after she was initially diagnosed with COVID-19. To investigate her SOB, a chest x-ray was performed in the ED, which showed right hemidiaphragmatic paralysis (Figure 1).

**Figure 2:**
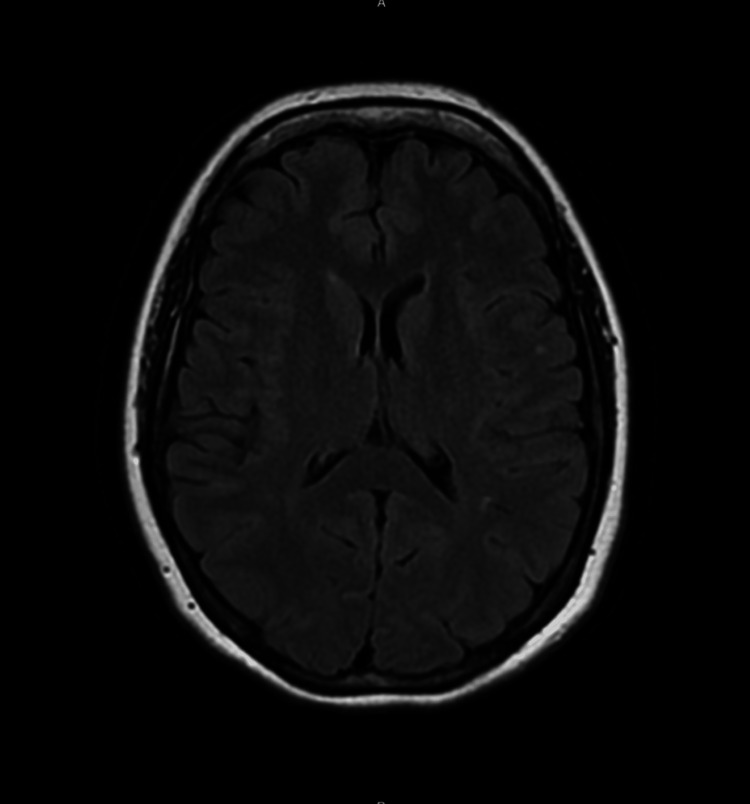
Axial T2-FLAIR MRI showing mild periventricular white matter changes and two punctate hyperintense lesions in the left hemispheric region. FLAIR: fluid attenuated inversion recovery

A brain magnetic resonance image (MRI) was only remarkable for non-specific scattered punctate white matter hyperintensities which did not explain this patient's symptoms (Figure 2). The patient was stabilized, discharged home, and referred to outpatient neurology.

The outpatient neurologist’s physical examination showed primarily unilateral symptoms with a right foot drop (3/5), right lower facial weakness, severe SOB, multiple decreased reflexes principally on the right, fatigable right-hand grip, wide-based and right foot steppage gait, numbness and tingling in the right upper and lower extremities, and bilateral arm strength of 4/5; however, she was able to get in and out of a chair with minimal assistance. This patient's right-hand grip strength was 1/5. Other examination findings included a right facial nerve palsy and otherwise intact cranial nerves (CN) II-VI and VIII-XII, a negative Hoffman’s and Lhermitte's sign, and decreased tone in all four extremities. The patient was negative for resting, intention, or action tremors; cerebellar function was intact, and no fasciculations were noted. Muscle tone was decreased in her right and left arms and legs. A sensory examination showed decreased vibration and pinprick sensation in a distal to proximal direction in both the right and left legs. Hypoactive reflexes included the right bicep, right and left brachioradialis, right patellar, and the right and left Achilles. All other reflexes tested were 2+/4. Labs were drawn for further testing of the etiology of this demyelinating neuropathy to rule out nutritional, inflammatory, autoimmune, endocrine, and infectious causes.

Laboratory tests including IgG and IgM anti-ganglioside-monosialic acid, anti-neoplastic, anti-nuclear, and anti-glutamic acid decarboxylase antibodies were negative (Table 1). Cerebral spinal fluid protein was only mildly elevated while creatine kinase, vitamin B12, acetylcholine receptor binding antibody, sedimentation rate, C-reactive protein, and thyroid-stimulating hormone were all within normal ranges. Laboratory analysis was significant for an elevated A1C of 7.1%.

**Table 1:** Laboratory values used to evaluate the causes of the patient's symptoms.

Investigations	Result	Reference range
IgG/IgM anti-ganglioside-monosialic acid	Negative	Negative
Anti-neoplastic antibody	Negative	Negative
Anti-nuclear antibody	Negative	Negative
Anti-glutamic acid decarboxylase antibodies	Negative	Negative
Cerebrospinal fluid protein	46 mg/dL	15-45 mg/dL
Creatine kinase	70 U/L	21-215 U/L
Vitamin B12	550 pg/mL	193-986 pg/mL
Acetylcholine receptor binding antibody	0.03 nmol/L	0.00-0.24 nmol/L
Sedimentation rate	7 mm/h	0-20 mm/h
C-reactive protein	0.5 mg/dL	0.0-0.9 mg/dL
Thyroid-stimulating hormone	2.250 mU/L	0.358-3.740 mU/L
Hemoglobin A1C	7.1%	4.8-5.6%

**Figure 3:**
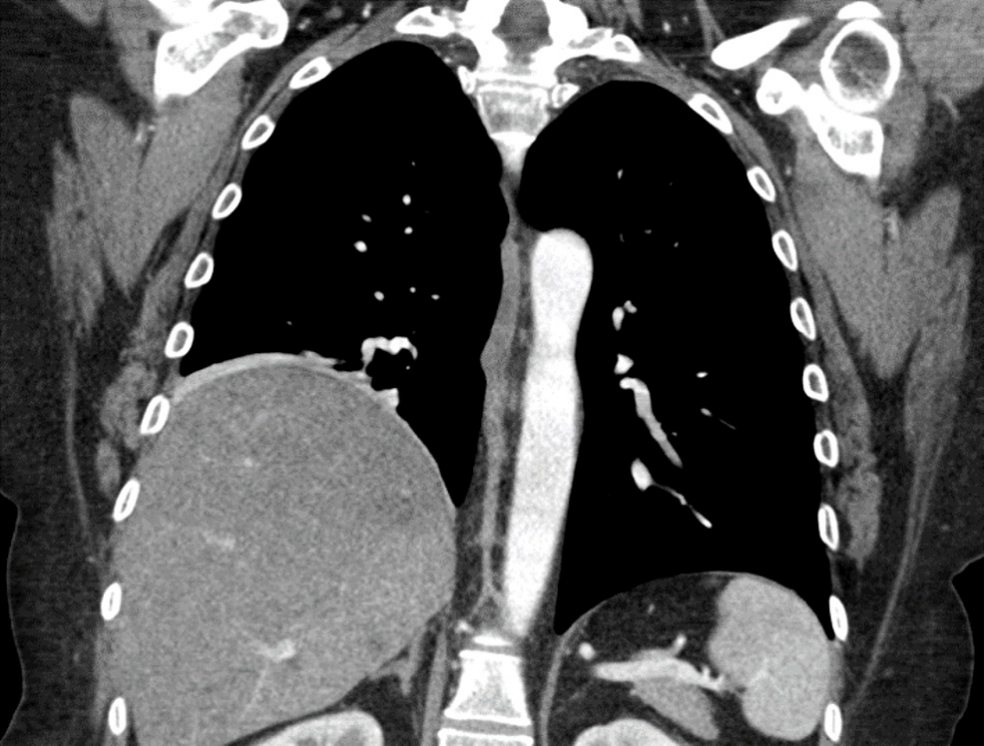
Coronal chest CT showing elevated right hemidiaphragm due to unilateral phrenic nerve palsy.

A chest computed tomography (CT) with contrast showed an elevated right hemidiaphragm with adjacent right basilar subsegmental atelectasis; this confirmed her paralyzed right hemidiaphragm, which was subsequently diagnosed as phrenic nerve palsy (Figure 3). Urgent nerve conduction and electromyography (EMG) studies showed significant distal axonal demyelinating sensorimotor polyneuropathy.

The sensory nerve conduction study showed a mild slowing of the right median sensory conduction velocity (Table 2). There was a mild slowing of the bilateral sural sensory conduction velocity and sensory nerve action potential with prolonged latencies. Transcarpal sensory nerve conduction testing showed that the median transcarpal speed was significantly reduced. The motor nerve conduction study revealed an abnormal distal right median motor conduction velocity with increased terminal latency (Table 3). There was a severe reduction in amplitudes of the right median nerve, right ulnar, right peroneal, and right tibial latencies, with prolonged terminal latencies and very small compound muscle action potentials with marked attenuation. The right ulnar nerve also showed slowing across the elbow. There was no response in the right peroneal F-waves, and the right tibial F-waves were mildly prolonged (Table 4). The right soleus H-reflex showed a mildly prolonged latency compared to the left (Table 5).

**Table 2:** Sensory nerve conduction study. ADM: abductor digiti minimi; Ant: anterior; APB: abductor pollicis brevis; AH: abductor hallucis; EDB: extensor digitorum brevis; Fib: fibular; Pop: popliteal; Tib: tibialis

Nerve/sites		Recorded site	Onset latency (ms)	Peak latency (ms)	Amplitude (µV)	Segments	Distance (mm)	Peak difference (ms)	Velocity (m/s)	Temperature (°C)	PP amplitude (µV)
R median - digit II (anti-dromic)	Wrist	Digit II	2.60	3.56	52.8	Wrist - digit II	130	-	50	31.3	54.6
R ulnar - digit V (antidromic)	Wrist	Digit V	2.02	2.92	50.7	Wrist - digit V	110	-	54	31.9	54.6
R radial - anatomical snuff box (forearm)	Forearm	Wrist	1.63	2.40	41.0	Forearm - wrist	100	-	62	31.1	43.5
R sural - ankle (calf)	Calf	Ankle	3.29	4.23	25.2	Calf - ankle	140	-	43	31.8	20.6
L sural - ankle (calf)	Calf	Ankle	2.60	3.44	31.0	Calf - ankle	140	-	54	31.3	35.7
R median, ulnar - transcarpal comparison	Median palm	Wrist	1.94	2.50	66.8	Median palm - wrist	80	-	41	31	67.9
	Ulnar palm	Wrist	1.29	1.75	46.9	Ulnar palm - wrist	80	-	62	31.2	47.3
	-	-	-	-	-	Median palm - ulnar palm		0.75	-	31.2	-

**Table 3:** Motor nerve conduction study. ADM: abductor digiti minimi; Ant: anterior; APB: abductor pollicis brevis; AH: abductor hallucis; Fib: fibular; Pop: popliteal; Tib: tibialis; L: left; R: right; A: above; B: below

Nerve/sites		Muscle	Latency (ms)	Amplitude (mV)	Duration (ms)	Segments	Distance (mm)	Latency difference (ms)	Velocity (m/s)	Temperature (°C)	Area (mVms)
R median - APB	Wrist	APB	5.17	3.0	5.25	Wrist - APB	70	-	-	31.8	8.6
	Elbow	APB	10.13	3.4	5.42	Elbow - wrist	250	4.96	50	31.8	9.4
L median - APB	Wrist	APB	4.02	10.7	6.06	Wrist - APB	70	-	-	32.4	37.8
	Elbow	APB	8.23	10.3	6.15	Elbow - wrist	230	4.21	55	32.4	34.8
R ulnar - ADM	Wrist	ADM	3.19	3.6	5.08	Wrist - ADM	70	-	-	31	8.5
	B elbow	ADM	7.38	2.8	5.77	B elbow - wrist	210	4.19	50	31.5	7.8
	A elbow	ADM	9.81	2.7	5.50	A elbow - B elbow	100	2.44	41	31	7.6
	-	-	-	-	-	A elbow - wrist	-	6.63	-	31	-
L ulnar - ADM	Wrist	ADM	2.83	12.8	6.50	Wrist - ADM	70	-	-	32.5	47.7
	B elbow	ADM	6.65	11.2	7.29	B elbow - wrist	210	3.81	55	32.4	45.3
	A elbow	ADM	8.50	11.1	6.92	A elbow - B elbow	100	1.85	54	32.4	43.3
	-	-	-	-	-	A elbow - wrist	-	5.67	-	32.4	-
R peroneal - EDB	Ankle	EDB	7.15	0.3	6.42	Ankle - EDB	80	-	-	31.8	1.2
	Fib head	EDB	13.15	0.2	7.21	Fib head - ankle	280	6.00	47	31.8	0.9
	Pop fossa	EDB	15.35	0.2	7.21	Pop fossa - Fib head	100	2.21	45	31.8	0.9
L peroneal - EDB	Ankle	EDB	4.60	6.3	7.17	Ankle - EDB	80	-	-	31.3	25.9
	Fib head	EDB	10.35	5.9	8.25	Fib head - ankle	300	5.75	52	31.4	25.1
	Pop fossa	EDB	12.21	5.7	7.25	Pop fossa - Fib head	100	1.85	54	23.9	22.0
R tibial - AH	Ankle	AH	11.17	0.4	5.94	Ankle - AH	80	-	-	31.3	1.5
	Pop fossa	AH	21.96	0.4	6.31	Pop fossa - ankle	420	10.79	39	31.1	1.4
L tibial - AH	Ankle	AH	5.90	4.1	4.94	Ankle - AH	80	-	-	31.2	8.9
	Pop fossa	AH	13.65	4.3	5.69	Pop fossa - ankle	410	7.75	53	31.2	12.0
R peroneal - Tib Ant	Fib head	Tib Ant	5.56	1.2	8.71	Fib head - Tib Ant	-	-	-	32.1	5.8
	Pop fossa	Tib Ant	7.27	1.5	8.62	Pop fossa - Fib head	100	1.71	59	32.2	6.6

**Table 4:** F-wave assessing for axonal polyneuropathy showing delayed or absent F-waves in the right peroneal and right tibial nerves. ADM: abductor digiti minimi; APB: abductor pollicis brevis; AH: abductor hallucis; EDB: extensor digitorum brevis; L: left; R: right; NR: no response; M: M-wave; F: F-wave

Nerve	F latency (ms)	M latency (ms)	F-M latency (ms)	F %	F estimate (ms)
R median - APB	30.4	4.9	25.5	100	-
R ulnar - ADM	29.0	3.1	25.8	50	-
R peroneal - EDB	NR	NR	NR	NR	NR
R tibial - AH	58.8	12.0	46.8	18.2	-
L peroneal - EDB	48.3	4.7	43.6	50	-
L tibial - AH	50.1	6.0	44.1	100	-
L median - APB	25.7	4.3	21.4	83.3	-
L ulnar - ADM	27.4	2.4	25.0	85.7	-

**Table 5:** H-reflex from bilateral soleus muscles. M: M-wave; H: H-reflex

Nerve	M latency, max M (ms)		M latency, max H (ms)		H latency (ms)	
Tibial	Left	Right	Left	Right	Left	Right
	6.41	7.45	6.41	-	19.84	36.41

The needle EMG findings revealed the right flexor digitorum superficialis and tibialis anterior (TA) had significant denervation, and the right extensor digitorium communis (EDC) showed moderate denervation (Table 6). The right gastrocnemius medial head indicated mild denervation. Recruitment of the right first dorsal interosseous, EDC, deltoid, TA, gastrocnemius, and vastus lateralis was moderate to low. The amplitudes of the muscles in the right lower extremity were significantly reduced. No myotonia or fasciculations were present. Repetitive nerve stimulation in the right abductor digiti minimi did not show decrement in compound muscle action potentials (Table 7). These findings were compatible with a significant distal axonal demyelinating sensorimotor polyneuropathy.

**Table 6:** Electromyography study showing denervation and a reduction in recruitment and amplitudes in the distribution of multiple individual peripheral nerves. R: right; N: normal; IA: insertional activity; Fib: fibrillations; PSW: polyspike wave; Fasc: fasciculations; HF: high frequency; Amp: amplitude; Dur: duration; MUAP: motor unit action potential; PPP: polyphasic potentials; DA: discrete pattern; RIP: reduced interference pattern; 1+, 2+, 3+, 4+: the degree of finding severity (1 is minimal, 4 is maximal)

Muscles, nerves, and nerve roots evaluated			Spontaneous					MUAP			Recruitment
Muscle	Nerve	Roots	IA	Fib	PSW	Fasc	HF	Amp	Dur	PPP	Pattern
R first dorsal interosseous	Ulnar	C8-T1	3+	4+	4+	None	None	N	N	N	Discrete
R extensor digitorum communis	Radial	C7-C8	3+	2+	3+	None	None	N	N	N	DA w/RIP
R deltoid	Axillary	C5-C6	N	None	None	None	None	N	N	N	Reduced
R tibialis anterior	Deep peroneal (fibular)	L4-L5	3+	4+	3+	None	None	1-	N	N	DA w/RIP
R gastrocnemius (medial head)	Tibial	S1-S2	1+	1+	1+	None	None	1-	N	N	Reduced
R vastus lateralis	Femoral	L2-L4	2+	None	None	None	None	1-	N	N	Reduced

**Table 7:** Repetitive nerve stimulation.

Anatomy/train		Rate (Hz)	Amplitude (mV)	Amplitude 4-1%
R abductor digiti minimi (manus) - (ulnar)				
Baseline @ 3 Hz		3	3.6	11.5
Post-exercise	@ 0:00	3	3.5	6.7
	@ 1:00	3	3.5	7.2
	@ 2:00	3	3.6	9.7
	@ 3:00	3	3.6	8.8

A diagnosis of MADSAM was made following extensive evaluation. This was believed to be an acquired autoimmune disease resulting from her previous COVID-19 infection eight months prior. The predominant unilateral symptoms, including right hemidiaphragmatic paralysis and right lower facial muscle weakness with multiple asymmetric sensory and motor nerves affected, along with the EMG results, made this presentation highly suspicious for the MADSAM variant of CIDP.

First-line treatments, including IVIG and intravenous (IV) corticosteroids, were discussed with the patient. The patient was started on IVIG treatment for long-term maintenance. An IVIG induction dose of 2 g/kg over five days was started, followed by monthly maintenance doses of 0.4 g/kg that can be administered and adjusted every three to four weeks based on the patient’s needs for a minimum of five years. At her three-month follow-up visit, her initial hypoactive reflexes, sensation in both legs, and facial weakness were mildly improved, and her right tibialis anterior muscle, responsible for her right foot drop, had improved to 4/5 strength; however, the phrenic nerve palsy had not improved. Both IVIG and corticosteroids (oral or IV) are first-line induction treatments for MADSAM [6,8]. Subcutaneous immunoglobulin (SCIg) may be used instead of IVIG for maintenance therapy, and plasma exchange has been shown to be an effective option when primary treatment fails [6-8]. Corticosteroids have been used for decades to treat CIDP, but they were avoided in this patient due to the potential long-term side effects, including weight gain, cushingoid appearance, hyperglycemia, peptic ulcer disease, insomnia, infection, cataracts, and osteoporosis [9].

## Discussion

Multifocal acquired demyelinating sensory and motor neuropathy, also called asymmetrical or multifocal CIDP or Lewis-Sumner syndrome, is a demyelinating mononeuritis multiplex and one of the most common atypical forms of CIDP. The preponderance of patients with MADSAM will present with mixed sensory and motor symptoms (>50%); however, patients may present with primarily motor (18%) or sensory (32%) symptoms [10]. The prevalence of typical CIDP is approximately 4.8-8.9 cases per 100,000 persons [4] while the multifocal variant is approximately 1-9 cases per 1,000,000 persons [3]. CIDP and its variants are very frequently misdiagnosed in clinical practice [11]. MADSAM classically presents as asymmetric large fiber neuropathy in a peripheral nerve distribution with asymmetric upper and lower motor and sensory dysfunction and an insidious onset that progresses over weeks to years [6].

Increasing evidence suggests that patients with diabetes have a higher prevalence of CIDP and its variants [12]. This patient has a history of chronically elevated A1C, suggesting diabetic radiculopathy; however, diabetic neuropathy manifests as small fiber neuropathy, so we believe this led to the patient only having an increased susceptibility to nerve damage. Due to the patient’s loss of vibration sense, gait ataxia, decreased reflexes, and paresthesia, as proven by the EMG and nerve conduction study, we believe this to be a large fiber neuropathy. Small fiber neuropathies; however, would manifest with impairment of pain, temperature, and autonomic functions, which are not present in this patient and could only be diagnosed with a skin biopsy.

Typical CIDP commonly presents with significantly elevated CSF protein due to its effects on proximal nerve segments. However, in MADSAM patients, approximately 82% have mild to moderately elevated CSF protein, with some MADSAM cases reporting a normal CSF protein level due to this neuropathy more commonly affecting the intermediate and distal nerve segments [10,13]. Typical CIDP also presents with symmetrical peripheral neuropathy and areflexia, unlike in our patient [7]. Over time, MADSAM can eventually present indistinguishably from typical CIDP as the disease progresses in severity to a more symmetric pattern [14]. In MADSAM, 48% of patients have CN involvement, with 80% having unilateral palsy, whereas in typical CIDP, only 11% have CN involvement [15]. Optic neuritis and other cranial nerve palsies such as oculomotor, trigeminal, and facial palsies have been reported in MADSAM patients [7]. We believe this to have caused the right lower facial droop in our patient, leading to her slurred speech.

Phrenic nerve palsy has been reported in as much as 20% of acute-onset typical CIDP patients [16]; however, there were no cases of phrenic nerve palsy specific to the MADSAM variant found in the literature. The pathophysiology behind this disease is not completely understood; however, the classical concept is that MADSAM neuropathy develops from macrophage-induced demyelination [17]. Further investigation may include an MRI and nerve biopsies [6]. These tests may show non-specific inflammation and patchy swelling in the nerve trunks and extensive onion bulb formations, which are strongly related to macrophage-induced demyelination [10,17].

MADSAM can also be difficult to diagnose due to its close symptomatic relationship to other forms of atypical CIDP, typical CIDP, and multifocal motor neuropathy (MMN). MMN, and all forms of CIDP, are chronic and progressive in nature. Also, MMN rarely has elevated CSF protein levels [13]. MADSAM can mimic MMN if it affects the upper motor nerves in an asymmetric way, but MADSAM is distinguished by the presence of sensory problems, does not have anti-GM1 antibodies, and does not cause fasciculations [7].

EMG findings in CIDP and its variants can include both full or partial conduction blocks, decreased amplitudes, slow conduction velocities, prolonged distal latencies, and delayed or absent F waves in one or more motor nerves, as presented in this patient [18]. MADSAM has also been described in the literature as having multiple conduction blocks in the intermediate nerve segment [18]. However, instead of multiple conduction blocks, this patient only had one conduction block located within the peroneal nerve.

COVID-19-associated neuromuscular diseases, including Guillain-Barré syndrome, are the second most prevalent neurological sequelae of this infection [19]. COVID-19 has been linked to a systemic inflammatory response and demyelinating neuropathies in the central and peripheral nervous systems; however, the pathologic basis of viral- or vaccine-related COVID-19 neuropathies is still unclear [20].

## Conclusions

This case is reported due to MADSAM’s rarity and to support the link between MADSAM and COVID-19 and the development of neurological disease post-infection. Increasing evidence suggests the emergence of autoimmune diseases following a COVID-19 infection. CIDP and its variants have not been consistently linked to a particular pathogen, but in this case, given the timeline of symptoms, her clinical presentation was most likely caused by an autoimmune reaction due to her prior COVID-19 infection.
